# Effect of Plate Thickness on the Laser-Welded Microstructure and Mechanical Properties of 22MnB5 Hot-Forming Steel

**DOI:** 10.3390/ma17246138

**Published:** 2024-12-15

**Authors:** Kaixuan Sang, Xin Li, Yu Zhang, Lingling Yi, Jing Chen, Yuting Lu, Zhengwei Gu

**Affiliations:** 1 Key Laboratory of Automobile Materials, School of Materials Science and Engineering, Jilin University, Changchun 130022, China; sangkaixuan2022@163.com (K.S.); yill20@mails.jlu.edu.cn (L.Y.); cj1540307503@163.com (J.C.); lyt6aha@163.com (Y.L.); 2 Beijing Ideal Automobile Corporation Limited, Changzhou Branch, Changzhou 213172, China; 18356472302@163.com

**Keywords:** 22MnB5, plate thickness, laser welding, microstructure, mechanical properties

## Abstract

To meet the development and application needs of advanced high-strength steel, the laser welding of 22MnB5 hot-forming steel plates with thicknesses of 2 mm, 3 mm, and 4 mm was studied in this paper. Mechanical testing revealed that as plate thickness increased, the tensile strength of welded joints decreased from 1489 MPa to 1357 MPa and 1275 MPa, equating to 96%, 91%, and 88% of the corresponding base metal strength, respectively. The heat-affected zone exhibited the lowest mechanical properties. Microstructural characterization showed that with increasing plate thickness, martensite grains in the welded joints grew larger, transitioning from fine acicular to larger island structures. Concurrently, dislocation density in the welded joints decreased gradually. Furthermore, microstructural changes in the heat-affected zone were more pronounced than those in the fusion zone. The larger grain size and reduced dislocation density softened the joint structure, which consequently decreased the strength and hardness of the welded joint. Laser-welded joints of three thicknesses can exceed 85% of the corresponding base metal strength, demonstrating strong industrial application potential.

## 1. Introduction

As environmental awareness rises and modern industry advances, the demand for lightweight and safer transportation vehicles continues to grow [1,2]. As an advanced ultra-high-strength steel, 22MnB5 hot-forming steel allows for reduced thickness while maintaining structural strength, achieving the goal of lightweight design. It has been widely used in load-bearing structural components such as A-pillars, B-pillars, and crash beams in automobiles [3,4,5]. The thickness of these components typically does not exceed 2 mm. Welding, a key process for permanently joining metallic materials, is essential to the mechanical performance of steel structures. In recent years, extensive research has been conducted on the welding performance of 22MnB5 hot-forming steel with thicknesses of 2 mm or less [6,7,8,9].

According to the reviewed literature, the welding methods for 22MnB5 steel with plate thicknesses of 2 mm and below include resistance spot welding [10,11,12], friction stir welding [13], electric arc welding [14], and laser welding [15,16,17]. Among these methods, laser welding stands out for its superior joint quality, with joint strength exceeding 85% of the base metal (BM). Laser welding is also characterized by high efficiency, a high degree of automation, and versatility across various welded structures, making it the mainstream welding method for high-strength steels [18,19,20,21]. Zhao et al. [22] conducted a study on butt-joint laser welding of 22MnB5 steel and QP980 steel, both with 1.2 mm plate thickness. They analyzed the effect of heat input on the microstructural evolution and mechanical properties of these dissimilar joints. They found that the welded heat-affected zone (HAZ) of the welded 22MnB5 hot-forming steel softened due to tempering. Kim et al. [23] studied splice welding of 22MnB5 steel with a plate thickness of 1.8 mm using a laser welding process. They obtained welded joints with a strength of about 85% of the BM and noted that implementing a rapid cooling strategy can effectively reduce the width of the HAZ, thereby minimizing softening. Aderibigbe et al. [24] conducted a study on laser stitching welding of 22MnB5 steel with a thickness of 1.8 mm and dual-phase steels (DP600, DP800, and DP1000), achieving welding joints with tensile properties comparable to those of the BM. The findings regarding the welding of 22MnB5 hot-forming steel with thicknesses of 2 mm or less have been widely applied in the automotive field.

With the wider adoption of 22MnB5 hot-forming steel, its use has expanded beyond the automotive industry to include special machinery manufacturing and military applications. These fields impose stricter standards on material structural strength, leading to the conclusion that 22MnB5 steel with a thickness of less than 2 mm fails to meet these requirements. Therefore, research on the welding process of 22MnB5 hot-forming steel with thicknesses of 2 mm or greater has become crucial.

In previous studies, Zhao et al. [25] used TIG welding to weld 22MnB5 before and after quenching with a plate thickness of 3 mm. They found that ferrite and pearlite in the unquenched material transformed into martensite after quenching. The quenched BM-welded joints exhibited good mechanical properties and a tensile strength of 1179.59 MPa, which is 73% of the quenched BM. Tomasz et al. [26] studied MAG welding on 22MnB5 steel pipes with a wall thickness of 3.6 mm. They obtained welded joints with good performance and analyzed the corresponding joint organization. Currently, welding 22MnB5 steel with thicknesses of 2 mm or greater primarily depends on arc welding. This is because welding thicker materials requires a significant heat input to ensure complete penetration and form a molten pool, a feature particularly suited to arc welding. However, the high heat input and energy distribution in arc welding result in a wide fusion zone (FZ) and large grain size in the HAZ, weakening the welded joints. Arc welding typically results in less than 80% of the strength of quenched BM, limiting its practical industrial applications. However, the growing use of high-power lasers offers the potential to overcome these limitations. Laser welding of 22MnB5 hot-forming steel with thicknesses of 2 mm and above is expected to become a significant research focus.

Therefore, the laser welding of 22MnB5 hot-forming steel with plate thicknesses of 2 mm, 3 mm, and 4 mm was studied in this paper. The influence of plate thickness on the mechanical properties and microstructure of welded joints was revealed through mechanical property testing and microstructure characterization of welded joints. This research provides more application basis for laser welding of hot-forming steel with thicknesses of 2 mm and above.

## 2. Materials and Methods

### 2.1. Materials

The initial materials for the tests were supplied uncoated 22MnB5 steel plates with plate thicknesses of 2 mm, 3 mm, and 4 mm, respectively, and the chemical compositions are shown in Table 1. The 22MnB5 steel, as a low-alloy high-strength steel, achieves its excellent performance due to its optimized chemical composition. Among them, the addition of manganese (Mn) significantly enhances the strength of the steel, while even a very small amount of boron (B) notably improves its hardenability [27,28].

The 22MnB5 plates, with thicknesses of 2 mm, 3 mm, and 4 mm, were cut into 150 mm × 75 mm small plates. The steel plates of different thicknesses were batch-processed using the direct hot-forming technique in a box-type high-temperature furnace (model SX-G03173). The plates were heated to 950 °C for 5 min to achieve complete austenitization [29]. They were then immediately transferred to a custom-made flat die with a circulating cooling water channel for hot pressing and pressure quenching, resulting in the transformation of the BM from austenite to martensite.

### 2.2. Experimental Methods

**Figure 1:**
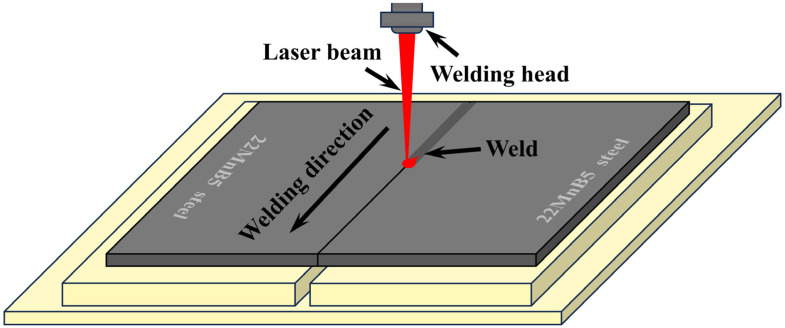
Schematic diagram of laser welding.

Laser welding was performed on hot-forming BM with plate thicknesses of 2 mm, 3 mm, and 4 mm, using butt joints as shown in Figure 1. Before welding, the surfaces were sequentially sanded with 300 # to 1200 # sandpaper and then cleaned with acetone to remove surface oils and oxides.

The laser model used for the welding test is the IPG-6kW YLR-U, operated with a KUKA robot for automation. The laser has a maximum power of 6 kW, a maximum welding speed of 100 mm/s, a wavelength of 1070 ± 10 nm, and an optical fiber diameter of 200 µm. Welding was conducted with 99.99% pure argon for gas protection, with a flow rate of 30 L/min. Based on the relevant literature [30] and pre-test results, the defocus amount for laser welding was set to 0, as this produces a weld with an optimal depth-to-width ratio. Welded joints with varying surface quality and mechanical properties were achieved by adjusting the laser power and welding speed. On the basis of several groups of welding test comparisons, three kinds of high-quality welded joints were identified. The laser power and welding speed of the 2 mm thick steel plate were determined to be 1.6 kW and 21 mm/s. When welding 3 mm and 4 mm thick steel plates, the welding speed and laser power were increased appropriately in order to reduce the adverse effects of high-temperature residence time on the joint microstructure. The welding speed was 55 mm/s, with laser powers set at 4.4 kW and 4.9 kW, respectively.

To assess the mechanical properties of welded joints under different welding parameters, the Vickers hardness of the specimens was measured using an automatic micro hardness tester model HVS-1000A. The indenter material is diamond. The testing parameters included a load of 500 g, a holding time of 10 s, and a test spacing of 0.1 mm. The tensile properties of the welded joints were evaluated using the DWD-50 electronic universal testing machine, which has a maximum tensile force of 50 kN and a tensile rate set at 2 mm/min. The strain was measured by an extensometer with a standard distance of 25 mm. To prevent cutting marks on the tensile specimens from affecting the test results, the cutting surfaces were polished with sandpaper prior to testing. For each welding parameter, three tensile specimens were tested, and the average maximum tensile strength of these specimens was used for characterization. Data with significant discrepancies were discarded, and new specimens were prepared for retesting.

Specimens measuring 6 mm × 4 mm × plate thickness were cut from the weld area using a cutting machine. The polished specimens were etched with a 4% nitric acid–alcohol solution for 10 s. After etching, the specimens were cleaned with alcohol and dried with air. The microstructure size and morphology of the specimens were observed using a tungsten filament scanning electron microscope, model TESCAN VEGA3-XMU. An alcohol solution containing 5% HClO_4_ was used as the electrolyte for electrolysis, with platinum electrodes as the electrodes. During electrolysis, the specimens were connected to the positive pole of the power supply, while the platinum electrodes were connected to the negative pole at a voltage of 30 V for 30 s. Liquid nitrogen was used to maintain the electrolyte temperature at 0 °C or below during the process. After electrolysis, the specimens were cleaned with alcohol and dried with air. Grain size, dislocation density, and recrystallization in the FZ and HAZ were statistically analyzed using a thermal field emission scanning electron microscope (SEM, JSM-7900F) with an electron backscattered diffraction (EBSD) probe.

To determine the phase composition of the material, the samples were sanded progressively with 600 # to 7000 # sandpaper to achieve a thickness of 100 μm or less. The thickness was then reduced to below 100 nm using ion thinning. After ion thinning, a transmission electron microscope (TEM, Talos F200X) was used to observe the microscopic morphology and to analyze the selected area electron diffraction of supplied-state BM, hot-forming BM, and welded FZ.

## 3. Results and Discussion

### 3.1. Microstructure and Properties of Base Metals Before and After Hot Forming

**Figure 2:**
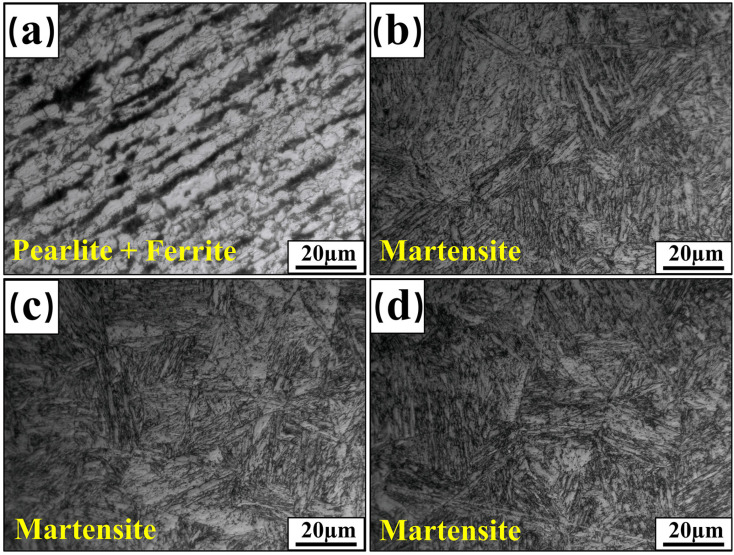
Microstructure of BM: (**a**) supplied state; (**b**) hot forming of 2 mm plate thickness; (**c**) hot forming of 3 mm plate thickness; (**d**) hot forming of 4 mm plate thickness.

The microstructural morphology of the BM before and after hot forming is shown in Figure 2. Figure 2a shows the microstructure of the 22MnB5 steel plates before hot forming, which mainly consists of lamellar pearlite and ferrite distributed in bands along the rolling direction. The microstructures of 22MnB5 steel plates with thicknesses of 2 mm, 3 mm, and 4 mm after hot forming are shown in Figure 2b–d, which are all transformed into martensite. Tensile tests were carried out on BM under two states respectively, and the tensile test results are shown in Table 2. The tensile tests reveal that the tensile strength of the supplied-state 22MnB5 steel plates before hot forming is 643 MPa, with an elongation of 16.41%, indicating good plasticity. The tensile strength of the BM after hot forming increased significantly, while elongation decreased markedly.

### 3.2. Mechanical Properties of Welded Joints

**Figure 3:**
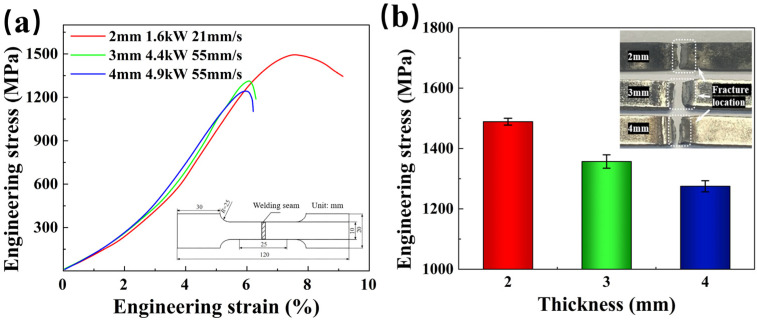
Tensile test of welded joints: (**a**) tensile curve; (**b**) tensile strength comparison.

The tensile curves and tensile strength trends of the specimens with three different plate thicknesses are shown in Figure 3. All specimens exhibited tensile fractures in the HAZ. The highest tensile strength of the welded joints, 1489 MPa, was found in the 2 mm plate thickness, reaching 96% of the corresponding hot-forming BM. As plate thickness increased, the tensile strength of specimens decreased to 1357 MPa and 1275 MPa. The tensile strength of the welded joints with 3 mm and 4 mm plate thicknesses were 91% and 88% of the corresponding hot-forming BM, respectively. Elongation decreased from 7.52% to 3.02% and 2.13% in the specimens. The significant decrease in elongation is attributed to stress concentration [31,32]. The tensile strength of the welded joints for all three plate thicknesses exceeded 85% of the corresponding hot-forming BM, indicating promising potential for industrial applications.

**Figure 4:**
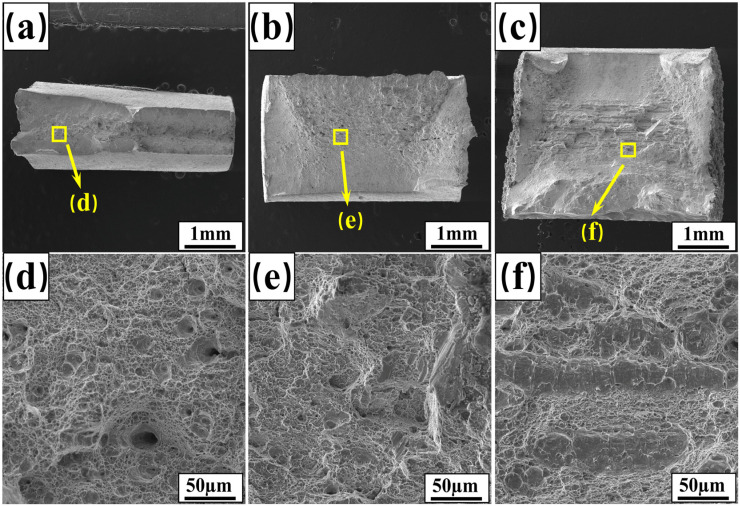
Fracture morphology of welded joints: 2 mm (**a**,**d**); 3 mm (**b**,**e**); 4 mm (**c**,**f**).

Figure 4 shows the tensile fracture morphology of welded joints. As shown in Figure 4a–c, fracture zones with a crystalline appearance are present in the fracture region of the specimens, indicating brittle fracture. This phenomenon is more pronounced in the samples with lower elongation values. Further localized magnification shows that the fracture of the specimen with a thickness of 2 mm contains many dimples of varying sizes (Figure 4d), indicating ductile fracture. As the specimen thickness increases, both the size and number of dimples decrease significantly (Figure 4e,f), while numerous river-like streaks appear, indicating quasi-cleavage fracture.

**Figure 5:**
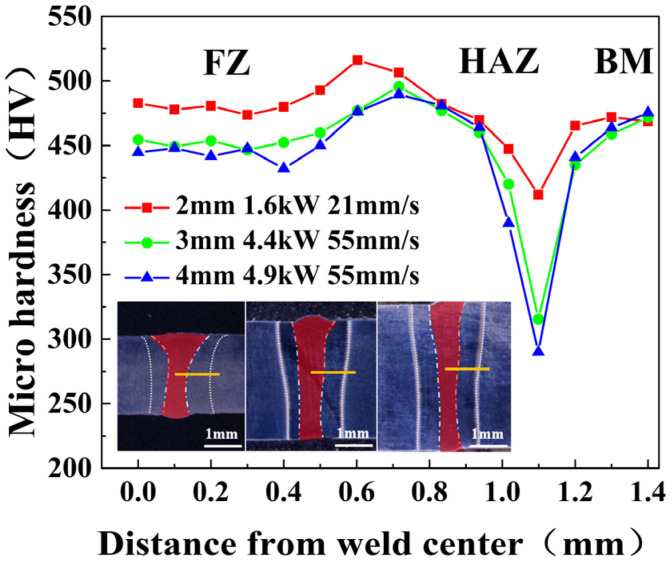
Comparison of microhardness of welded joints.

The microhardness distribution of the welded joints is shown in Figure 5. Analysis shows that the average microhardness of the FZ and HAZ with the 2 mm plate thickness is the highest, and the overall hardness fluctuation is relatively small. As the plate thickness increased, the average microhardness of the FZ and HAZ gradually decreased. The minimum microhardness values in the welded joints, 422 HV, 315 HV, and 291 HV, were observed in the HAZ. The HAZ, being the weakest area in terms of mechanical properties, is the first to fracture during tensile testing.

### 3.3. Microstructure of Welded Joints

**Figure 6:**
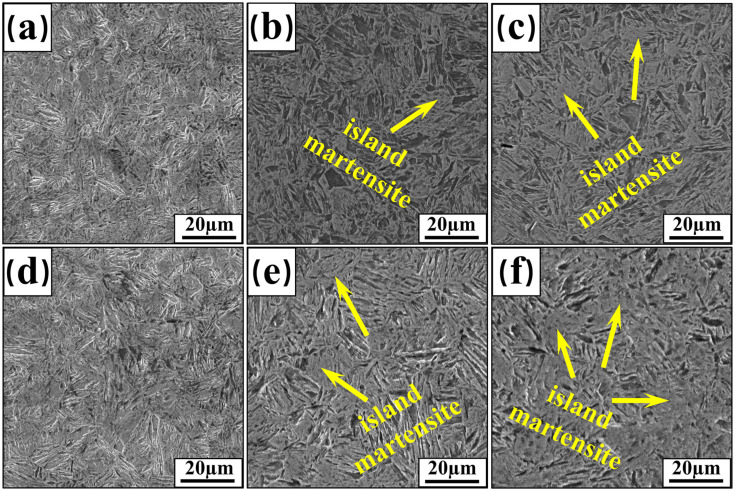
SEM images of welded-joint FZ and HAZ: 2 mm (**a**,**d**); 3 mm (**b**,**e**); 4 mm (**c**,**f**).

Figure 6 shows the SEM images of three types of welded-joint FZ and HAZ. The difference in grain size of welded joints with different plate thicknesses is relatively obvious. The 2 mm welded joint’s FZ and HAZ regions contain primarily fine acicular martensite. The grains are relatively small, around 10 μm, with minimal size variation. As plate thickness increased, martensite size in the welded joints grew significantly. Grain growth was more pronounced in the HAZ than in the FZ. Additionally, the morphology of martensite in welded joints also changed. As shown in Figure 6a,d, the martensite of both FZ and HAZ with 2 mm plate thickness showed a fine lath-like or acicular shape. However, the FZ and HAZ microstructure of welded joints with 3 mm and 4 mm thicknesses consisted of acicular martensite and a large amount of island martensite, and the area of island martensite increased with increasing plate thickness.

Analysis shows that as plate thickness increases, higher heat input and lower cooling rate promote the growth of austenite grains in the joint microstructure. Martensite forms through the transformation of austenite under non-equilibrium conditions. Larger austenite grains result in a reduction in grain boundary numbers, thereby reducing the nucleation sites for martensite. Consequently, both the amount of martensite and its formation sites decrease, while the tendency to form island martensite increases. When the cooling rate is slower, more heat is transferred to the HAZ and the grains have more time to grow, which results in further grain growth. During grain growth, the number of grain boundaries and dislocation density will also decrease, weakening the strengthening effect of the joint microstructure, which in turn reduces the strength and hardness of the welded joint. 

As plate thickness and heat input increase, larger grain sizes inevitably form in welded joints. The grain morphology gradually shifts from acicular to island-like. Simultaneously, the dislocation density decreases. As a result, the welded joint softens. This negative effect becomes more pronounced as plate thickness increases.

### 3.4. EBSD Analysis of Welded Joints

To investigate the effects of different plate thicknesses on grain size, dislocation density, and recrystallization of the welded joints, the samples were characterized using EBSD.

#### 3.4.1. Fusion Zone

**Figure 7:**
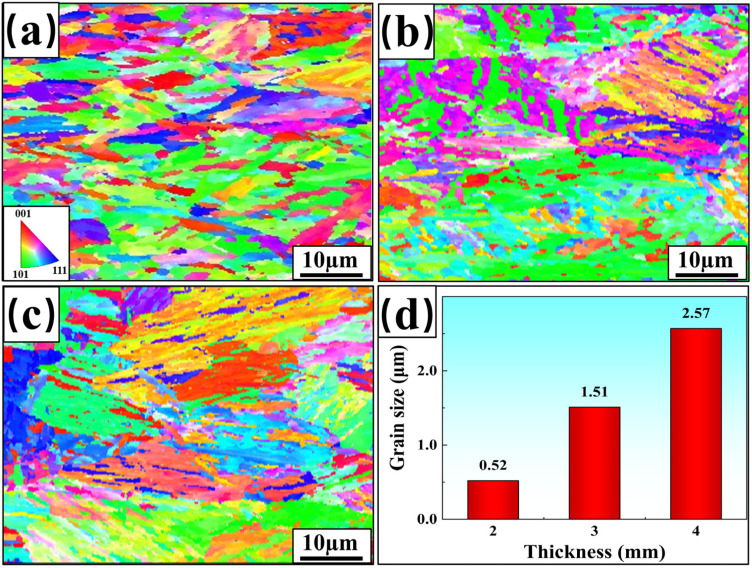
IPF and average grain size for welded-joint FZ: (**a**) 2 mm; (**b**) 3 mm; (**c**) 4 mm; (**d**) grain size statistics.

The Inverse Pole Figure (IPF) and average grain size statistics of the welded-joint FZ for three plate thicknesses are shown in Figure 7. Different colors in the figure represent different spatial orientations of the grains, with red indicating the direction [001], green indicating the direction [101], and blue indicating the direction [111]. Columnar crystal zones are observed in the FZ, formed as solidification progresses from the edges to the center, with grains growing in the direction of the thermal gradient [22]. Statistical analysis shows that the average grain sizes of FZ in welded joints with thicknesses of 2 mm, 3 mm, and 4 mm are 0.52 μm, 1.51 μm, and 2.57 μm, respectively. The grain size of FZ becomes larger with the increase in plate thickness. The effect of fine-grain strengthening gradually weakened, reducing the mechanical properties of welded joints. This is consistent with the previous analysis.

**Figure 8:**
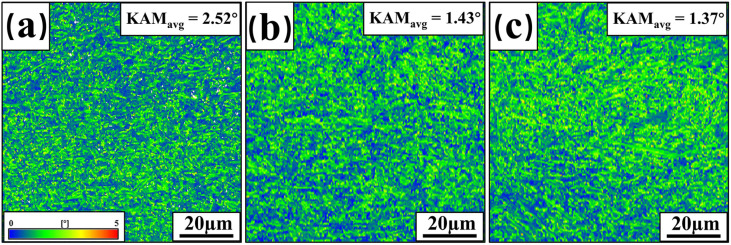
KAM of welded-joint FZ: (**a**) 2 mm; (**b**) 3 mm; (**c**) 4 mm.

**Figure 9:**
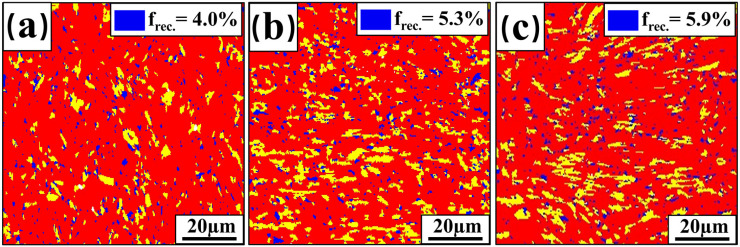
Recrystallisation of welded-joint FZ: (**a**) 2 mm; (**b**) 3 mm; (**c**) 4 mm.

To gain a comprehensive understanding of the microstructural changes in the welded FZ, the samples with different thicknesses were further characterized. Figure 8 displays the Kernel Average Misorientation (KAM) maps for the FZ of three thicknesses of welded joints. KAM is a method used in EBSD data analysis to characterize local misorientation angles and is also used to estimate the dislocation density in samples [33]. KAM is positively correlated with the density of geometrically necessary dislocations (GNDs) generated by phase transformations [34]. Blue regions indicate lower GND densities, while red and green regions represent higher GND densities [35]. According to the statistics, the KAM values of the welded FZ for 2 mm, 3 mm, and 4 mm thick plates are 2.52°, 1.43°, and 1.37°, respectively. Dislocation density decreases with increasing plate thickness, which reduces the mechanical properties of welded joints. Figure 9 shows the recrystallization ratios of three welded-joint FZs. In this figure, blue represents recrystallized grains (with orientation differences of 0~2°), yellow denotes subgrains (with orientation differences ranging from 2° to 7.5°), and red indicates deformed grains (with orientation differences greater than 7.5°). The recrystallization ratios (f_rec._) of the welded-joint FZ are 4.0%, 5.3%, and 5.9% for 2 mm, 3 mm, and 4 mm plate thicknesses, respectively. 

This indicates that higher temperatures with lower cooling rates promote recrystallization as the plate thickness and welding heat input increase. Recrystallization involves grain boundary migration, which reduces dislocation density. This finding is consistent with the KAM map analysis. As dislocation density decreases, the resistance of the welded FZ to deformation also decreases, resulting in a gradual reduction in the strength and hardness of the welded joints.

#### 3.4.2. Heat-Affected Zone

Under the action of the laser heat source, the HAZ is affected by the welding thermal cycle and the microstructure will change. Moreover, HAZ is often the location where fracture occurs in welded joints. Therefore, the HAZ of welded joints is analyzed. 

**Figure 10:**
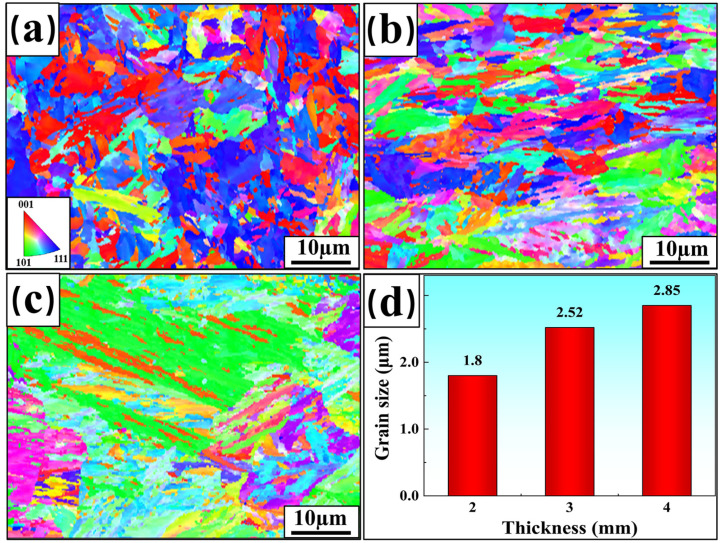
IPF and average grain size for welded-joint HAZ: (**a**) 2 mm; (**b**) 3 mm; (**c**) 4 mm; (**d**) grain size statistics.

The IPF and average grain size statistics for the HAZ of the three welded joints are shown in Figure 10. The figure shows that each zone is composed of coarse grains and some finer grains. The presence of coarse grains increases the average grain size. The statistics show that the average grain sizes of HAZ for joints with plate thicknesses of 2 mm, 3 mm, and 4 mm are 1.8 μm, 2.52 μm, and 2.85 μm, respectively. It is clear that the size of grain growth is positively correlated with plate thickness. The average grain size of the welded HAZ is coarser than that of the corresponding FZ. The larger grain size reduces the mechanical properties of the welded joints. This is one of the main reasons why tensile fracture usually occurs in HAZ.

**Figure 11:**
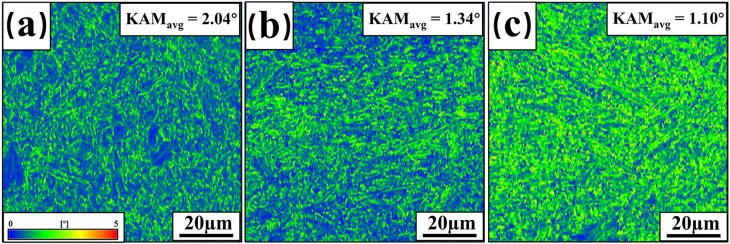
KAM of welded-joint HAZ: (**a**) 2 mm; (**b**) 3 mm; (**c**) 4 mm.

**Figure 12:**
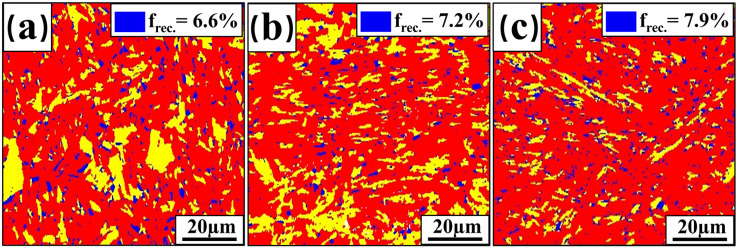
Recrystallisation of welded-joint HAZ: (**a**) 2 mm; (**b**) 3 mm; (**c**) 4 mm.

Figure 11 shows the KAM maps for the HAZ of the three welded joints. Statistically, the KAM values are 2.04°, 1.34°, and 1.10°, respectively, with the increase in plate thickness. It shows that the dislocation density of HAZ decreases with increasing plate thickness, which is the same trend as the dislocation density of FZ. Similarly, the recrystallization of HAZ changes with increasing heat input. Figure 12 shows the recrystallization ratio of HAZ for the three welded joints. The statistics show that the percentage of recrystallized HAZ in welded joints with 2 mm, 3 mm, and 4 mm plate thicknesses are 6.6%, 7.2%, and 7.9%, respectively. This indicates that the number of recrystallized grains in the HAZ increases with the increase of plate thickness, and, concurrently, the dislocation density decreases. In addition, the dislocation density in the HAZ decreases to smaller values with increasing plate thickness compared to the welded FZ, which is another main reason for tensile specimen fracture in the HAZ.

### 3.5. Phase and Structure Analysis

**Figure 13:**
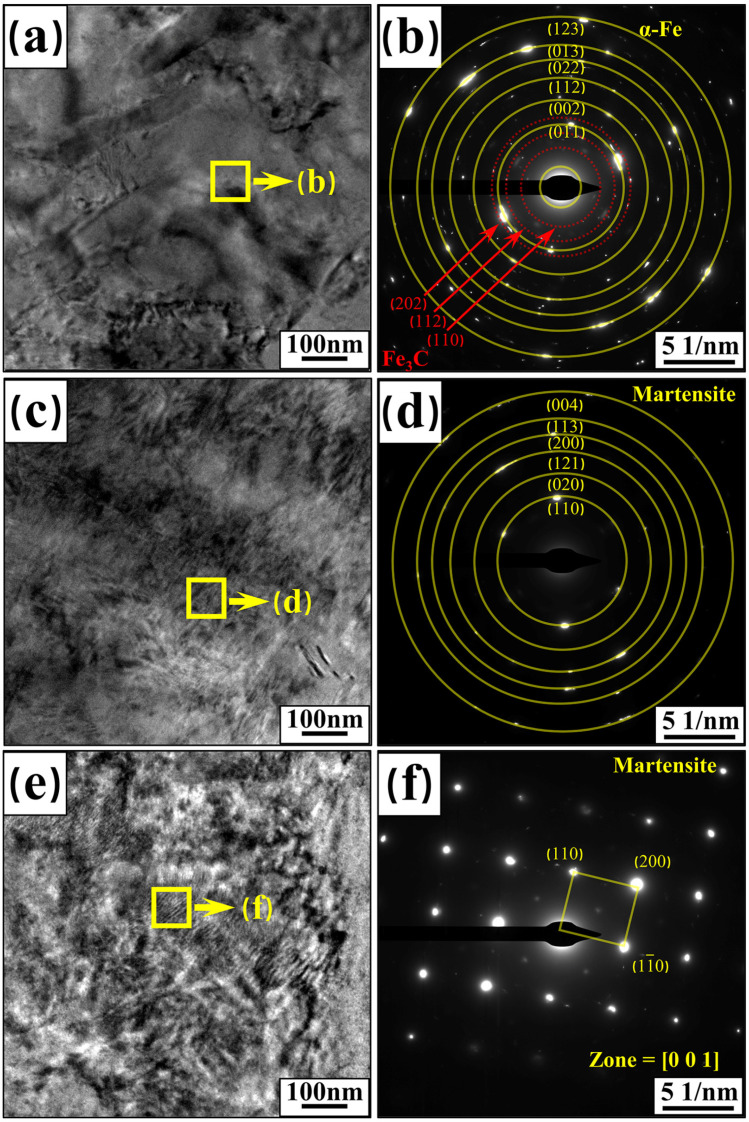
TEM images of supplied-state BM, hot-forming BM, and welded-joint FZ: (**a**,**b**) are the bright-field phase and selected-area electron diffraction pattern of the supplied-state BM; (**c**,**d**) are the bright-field phase and selected-area electron diffraction pattern of the hot-forming BM; (**e**,**f**) are the bright-field phase and selected-area electron diffraction pattern of the welded FZ.

In order to determine the microstructural composition of 22MnB5 steel plates, the corresponding samples were characterized and analyzed for their microstructure. TEM images of supplied-state BM, hot-forming BM, and typical welded-joint FZ are shown in Figure 13.

Analysis of Figure 13a reveals that the supplied-state BM exhibits a pearlitic matrix with a small amount of ferrite dispersed within it. This observation is corroborated by the electron diffraction pattern in Figure 13b, which identifies the α-Fe phase and Fe_3_C phase.

Figure 13c shows the bright field phase of the BM after hot forming. Analysis indicates that pearlite and ferrite in the BM have been transformed into acicular martensite, resulting in a fully martensitic structure. Figure 13d presents the electron diffraction pattern of the BM after hot forming, confirming a martensitic phase with no other phases present, and indicating a polycrystalline diffraction pattern. This suggests that the martensite grains are small.

Figure 13e shows the bright field phase of the welded-joint FZ. Compared to Figure 13c, the martensite size in the FZ has increased, with a small amount of acicular martensite interspersed among the island martensite. Figure 13f presents the electron diffraction pattern of the welded FZ, confirming the martensite phase. Unlike the hot-forming BM, which shows a polycrystalline diffraction pattern, the FZ exhibits a single-crystal diffraction pattern. This indicates that the martensite grains in the FZ are larger than those in the hot-forming BM. This is primarily because the hot-forming BM is cooled using water-cooled molds, resulting in a faster cooling rate and smaller martensite grains. In contrast, the welded FZ cools in air, where heat dissipation to the BM on both sides of the molten pool results in a slower cooling rate and more time for martensite growth. Consequently, the martensite grains in the welded FZ are larger than those in the hot-forming BM.

### 3.6. Microstructure Evolution

**Figure 14:**
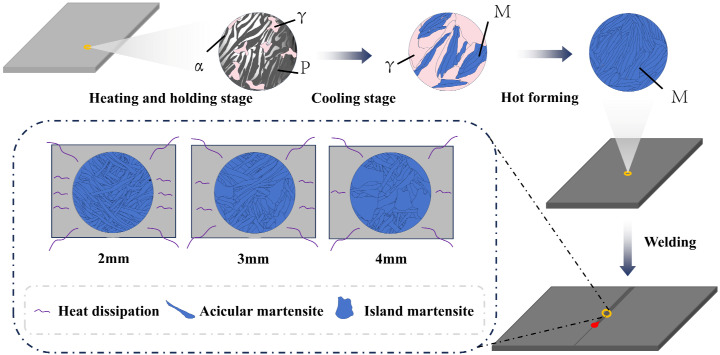
Microstructure evolution of 22MnB5 hot forming steel and welded joints.

Figure 14 shows the microstructure evolution of 22MnB5 during hot forming and welding. In the heating and holding stage of hot forming, austenite precipitates preferentially at the grain boundary between pearlite and ferrite and grows gradually [36]. Subsequent cooling conditions reached a critical cooling rate and martensite was rapidly generated within the austenite crystals. Since the three thicknesses of steel plates were subjected to the same hot-forming conditions, there were some differences in the microstructure and morphology of the martensite. Martensite and austenite have good interfacial coherence, which reduces the driving force required for interfacial migration [37]. Therefore, the hot-forming microstructure will have an effect on the subsequent microstructure of the welded joint.

As shown in Figure 6b,c,e,f, two distinct martensite morphologies, acicular and insular, appear in the welded-joint microstructure. A similar phenomenon was found by Fang et al. [15]. This can be explained by the martensite and austenite formation process. Smaller martensite grain sizes result in higher boundary densities, offering more nucleation sites for the austenite transformation [38]. Smaller austenite grain sizes favor the generation of finer-sized acicular martensite. During welding, in the FZ and HAZ zones, the grain size is strongly influenced by the cooling conditions. The increase in plate thickness results in a longer high-temperature residence time at the joint, reducing the cooling rate. Under smaller subcooling conditions, the grains tend to grow further. Concurrently, along with the annihilation and regeneration of dislocations, the tissue morphology is preserved during the subsequent cooling process.

The above analysis shows that grain refinement before welding is conducive to improving the mechanical properties of welded joints. During welding, rapid cooling of the molten pool can effectively alleviate the decrease in mechanical properties as plate thickness increases.

## 4. Conclusions

In this paper, 22MnB5 hot-forming steel plates were prepared by the quenching process. Laser welding tests were carried out on steel plates of 2 mm, 3 mm, and 4 mm thicknesses, respectively. The mechanical properties and microstructure of the three thicknesses of welded joints were studied. Based on the analysis and discussion of experimental results, the following conclusions were obtained.

1. The strength and hardness of the welded joints decreased gradually as plate thickness increased. The tensile strength decreased from 1489 MPa to 1357 MPa and 1275 MPa. The HAZ exhibited the lowest mechanical properties. This reduction in joint properties can be attributed to the increase in martensite grain size and the decrease in dislocation density.
2. SEM and EBSD analysis showed that as plate thickness increased, fine acicular martensite was gradually replaced by larger island martensite in the welded joints. The dislocation density in the martensite microstructure gradually decreased. Microstructural changes in the HAZ were more pronounced than those in the FZ. TEM analysis confirmed the transformation of the martensite microstructure. The increase in martensite size and the decrease in dislocation density softened the welded joints, resulting in a reduction in their mechanical properties.
3. The tensile strength of the laser-welded joints for three plate thicknesses all exceeded 85% of the corresponding BM strength, showing promising potential for industrial applications. This paper provided a reference for further improving the mechanical properties of welded joints.

## Figures and Tables

**Table 1:** Chemical composition of 22MnB5 steel (wt.%).

Element	C	Mn	Si	S	P	Al	B	Fe
wt.%	0.22	1.22	0.23	0.0018	0.022	0.0456	0.0033	Bal.

**Table 2:** Tensile test results of base metals.

Processing State of BM	Tensile Strength (MPa)	Elongation (%)
Supplied state	643 ± 13	16.41 ± 1.61
Hot forming (2 mm)	1552 ± 7	9.58 ± 0.73
Hot forming (3 mm)	1482 ± 7	9.77 ± 0.55
Hot forming (4 mm)	1444 ± 8	9.92 ± 0.64

## Data Availability

Datasets generated and/or analyzed during the current study are available from the corre-sponding author on request due to the lab's policies or confidentiality agreements.

## References

[1] Bok H.H., Lee M.G., Pavlina E.J., Barlat F., Kime H.D. (2011). Comparative study of the prediction of microstructure and mechanical properties for a hot-stamped B-pillar reinforcing part. Int. J. Mech. Sci..

[2] Zhang P.Y., Zhu L., Luo S.Y., Luo J.T. (2019). Hot stamping forming and finite element simulation of USIBOR1500 high-strength steel. Int. J. Adv. Manuf. Technol..

[3] Ximenes D.A.D.C., Moreira L.P., Carvalho J.E.R.D., Leite D.N.F., Toledo R.G., Dias F.M.D.F. (2020). Phase transformation temperatures and Fe enrichment of a 22MnB5 Zn-Fe coated steel under hot stamping conditions. J. Mater. Res. Technol..

[4] Peng W.J., Wu G.X., Zhang J.Y. (2020). In-situ observation of liquid zinc-induced erosion behavior diffusion mechanism in zinc-coated 22MnB5 steel. J. Mater. Res. Technol..

[5] Rossini M., Spena P.R., Cortese L., Matteis P., Firrao D. (2015). Investigation on dissimilar laser welding of advanced high strength steel sheets for the automotive industry. Mater. Sci. Eng. A.

[6] He X., Qin Y.Q., Jiang W.X. (2019). Effect of welding parameters on microstructure and mechanical properties of laser welded Al-Si coated 22MnB5 hot stamping steel. J. Mater. Process. Technol..

[7] Tuncel O., Aydin H., Davut K. (2023). Effect of heat input on HAZ softening in fiber laser welding of 22MnB5 steel. Opt. Laser Technol..

[8] Šebestová H., Horník P., Mikmeková Š., Mrňa L., Doležal P., Novotný J. (2021). Microstructural Characterization of Laser Weld of Hot-Stamped Al-Si Coated 22MnB5 and Modification of Weld Properties by Hybrid Welding. Materials.

[9] Wolf C., Völkers S., Kryukov I., Graß M., Sommer N., Böhm S., Wunder M., Köhler N., Mäckel P. (2022). Enhancement of weldability at laser beam welding of 22MnB5 by an entrained ultrasonic wave superposition. Materials.

[10] Jong Y.S., Lee Y.K., Kim D.C., Kang M.J., Hwang I.S., Lee W.B. (2011). Microstructural evolution and mechanical properties of resistance spot welded ultra high strength steel containing boron. J. Mater. Process. Technol..

[11] Kaars J., Mayr P., Koppe K. (2016). Generalized dynamic transition resistance in spot welding of aluminized 22MnB5. Mater. Des..

[12] Zhang K.P., Wu L.J., Tan C.W., Sun Y.M., Chen B., Song X.G. (2019). Influence of Al-Si coating on resistance spot welding of Mg to 22MnB5 boron steel. J. Mater. Process. Technol..

[13] Li T.T., Xie X.L., Xu J.F., Li R.F., Qi K., Zhang X.Q., Yue H.G., Zhao Y., Qiao L. (2023). Research on AZ31 Mg alloy/22MnB5 steel pinless friction stir spot welding process and interfacial temperature field simulation. J. Mater. Res. Technol..

[14] Fan H.J., Liu P., Xiao K., Wu C.G., Shi C.W., Wang Y.B. (2023). Microstructure and properties of pulse tungsten inert gas welded joint for different thickness CR22MnB5/DH1050 dissimilar high-strength steel. J. Mater. Res. Technol..

[15] Fang X., Wu Y.X., Yang X.Y., Yang Y.G., Cheng L., Zhang Q., Liu X.Y., Mi Z.L. (2024). Microstructure and mechanical properties of the laser welded air-hardening steel joint. Mater. Charact..

[16] Sun Y.M., Wu L.Y., Tan C.W., Zhou W.L., Chen B., Song X.G., Zhao H.Y., Feng J.C. (2020). Influence of Al-Si coating on microstructure and mechanical properties of fiber laser welded and then press-hardened 22MnB5 steel. Mater. Sci. Eng. A.

[17] Razmpoosh M.H., Macwan A., Biro E., Chen D.L., Peng Y., Goodwin F., Zhou Y. (2018). Liquid metal embrittlement in laser beam welding of Zn-coated 22MnB5 steel. Mater. Des..

[18] Sharma R., Molian P., Peters F. (2010). Geometric variability and surface finish of weld zones in Yb:YAG laser welded advanced high strength steels. J. Manuf. Process..

[19] Oyyaravelu R., Kuppan P., Arivazhagan N. (2016). Metallurgical and mechanical properties of laser welded high strength low alloy steel. J. Adv. Res..

[20] Wang J.F., Yang L.J., Sun M.S., Liu T., Li H. (2016). Effect of energy input on the microstructure and properties of butt joints in DP1000 steel laser welding. Mater. Des..

[21] Ramesh R., Dinaharan I., Ravikumar R., Akinlabi E.T. (2020). Microstructural characterization and tensile behavior of Nd:YAG laser beam welded thin high strength low alloy steel sheets. Mater. Sci. Eng. A.

[22] Zhao H.Y., Huang R.G., Sun Y.M., Tan C.W., Wu L.J., Chen B., Song X.G., Li G.X. (2020). Microstructure and mechanical properties of fiber laser welded QP980/press-hardened 22MnB5 steel joint. J. Mater. Res. Technol..

[23] Kim C.H., Choi J.K., Kang M.J., Park Y.D. (2010). A study on the CO_2_ laser welding characteristics of high strength steel up to 1500 MPa for automotive application. J. Achiev. Mater. Manuf. Eng..

[24] Aderibigbe I.A., Popoola P.A., Sadiku R.E., Biro E. (2022). Effects of heat input on microstructure and mechanical properties of dissimilar laser-welded dual-phase and boron steel joints. SAE Int. J. Mater. Manuf..

[25] Zhao H.Y., Liu H.W. (2014). Microstructure and properties of TIG welded 22MnB5 ultra high strength steel. Trans. China Weld. Inst..

[26] Pfeifer T., Weglowski M.S. (2014). Characteristic of MAG welded joints of 22MnB5 steel grade for automotive industry. Adv. Manuf..

[27] Li B.B., Chen Y., Zhao X.L., Li J.K., Wang C., Wang M.Q. (2023). Effect of austenitization process on oxidative decarbonization behavior and mechanical properties of 22MnB5 steel. J. Mater. Eng. Perform..

[28] Pan H.J., Wei C.F., Yu W.W., Li X.Y., Qiao B. (2022). Achieving excellent strength–ductility combination by cyclic quenching treatment in 22MnB5 steel. J. Mater. Eng. Perform..

[29] Çavuşoğlu O., Çavuşoğlu O., Yilmazoğlu A.G., Üzel U., Aydın H., Güral A. (2020). Microstructural features and mechanical properties of 22MnB5 hot stamping steel in different heat treatment conditions. J. Mater. Res. Technol..

[30] Zhang G.L., Kong H., Zou J.L., Zhao Z.J., Xiao R.S. (2021). Spatter characteristics of high-power fibre laser deep penetration welding and effect of defocus on spatter. Chin. J. Lasers.

[31] Xu W., Tao W., Luo H.W., Yang S.L. (2022). Effect of welding speed on microstructure and mechanical behavior of laser welded Al-Si coated 22MnB5 steel. Opt. Laser Technol..

[32] Teng F., Huan P.C., Wang X.N., Xu Q.S., Sun Q., Zhang Q.G., Lv D., Di H.S. (2024). Analysis on surface morphology formation process of Al-Si coated 22MnB5 steel during laser welding. Opt. Laser Technol..

[33] Liu X.Y., Sun Y.P., Li W.Z., He J.M., Pei M.Y., Zhang K.F. (2024). Effect of welding current on the organization and properties of welded joints of Q690D high-strength steel. Mater. Today Commun..

[34] Fang J.X., Ma G.Z., Tian H.L., Li S.B., Huang H.S., Liu Y., Jiang Y.L., Liu B. (2021). Transformation-induced strain of a low transformation temperature alloy with high hardness during laser metal deposition. J. Manuf. Process..

[35] Bhanu V., Malakar A., Guota A., Pandey C. (2023). Electron beam welding of P91 steel and incoloy 800HT and their microstructural studies for advanced ultra super critical (AUSC) power plants. Int. J. Press. Vessel. Pip..

[36] Gao B., Hu R., Pan Z.Y., Chen X.F., Liu Y., Xiao L.R., Cao Y., Li Y.S., Lai Q.Q., Zhou H. (2021). Strengthening and ductilization of laminate dual-phase steels with high martensite content. J. Mater. Sci. Technol..

[37] Zhang X.M., Mu Y., Dodaran M., Shao S., Moldovan D., Meng W.J. (2018). Mechanical failure of CrN/Cu/CrN interfacial regions under tensile loading. Acta Mater..

[38] Cheng P., Hu B., Liu S.L., Guo H., Enomoto M., Shang C.J. (2019). Influence of retained austenite and Cu precipitates on the mechanical properties of a cold-rolled and intercritically annealed medium Mn steel. Mater. Sci. Eng. A.

